# Supplementary material for: Detailed molecular and epigenetic characterization of the pig IPEC-J2 and chicken SL-29 cell lines
Source: iScience. 2023 Feb 20;26(3):106252. doi: 10.1016/j.isci.2023.106252 (PMC10018572; doi:10.1016/j.isci.2023.106252)
Supplement: Data S2. Complete homer output for identified motifs in Chicken SL-29, related to Tables 5 and 6 — Homer motif analysis results for histone modifications H3K4me1, H3K4me3, H3K27ac, enhancers, and ATAC-seq of chicken SL-29 cell line. Parameters for possible false positives is as mentioned earlier for S5. [file mmc3.zip › Data_S2/S6/Chicken_SL_29/peak_files_CTCF/homerResults/motif14.similar.html]

motif14

## Information for motif14

A
T
G
C
C
T
G
A
A
G
T
C
A
C
T
G
C
G
A
T
A
C
T
G
C
T
G
A
A
G
T
C
  
Reverse Opposite:  

A
C
T
G
A
G
C
T
A
G
T
C
C
G
T
A
A
G
T
C
C
T
A
G
A
G
C
T
A
T
C
G
  

|  |  |
| --- | --- |
| p-value: | 1e-53 |
| log p-value: | -1.235e+02 |
| Information Content per bp: | 1.890 |
| Number of Target Sequences with motif | 1752.0 |
| Percentage of Target Sequences with motif | 5.94% |
| Number of Background Sequences with motif | 1177.1 |
| Percentage of Background Sequences with motif | 4.05% |
| Average Position of motif in Targets | 152.8 +/- 83.9bp |
| Average Position of motif in Background | 149.4 +/- 90.6bp |
| Strand Bias (log2 ratio + to - strand density) | 0.0 |
| Multiplicity (# of sites on avg that occur together) | 1.05 |
| Motif File: | file (matrix) reverse opposite |

### Similar de novo motifs found

|  |  |  |  |  |  |  |  |
| --- | --- | --- | --- | --- | --- | --- | --- |
| Rank | Match Score | Redundant Motif | P-value | log P-value | % of Targets | % of Background | Motif file |
| 1 | 0.729 | T G C A G C A T G T A C C G A T A G T C A C T G A G T C A C T G G T C A A T C G | 1e-38 | -88.550221 | 1.62% | 0.84% | motif file (matrix) |
| 2 | 0.834 | A T C G A T C G C A G T T C A G G A C T T A G C G C T A T A G C A T C G A C G T A C T G T C G A A T G C | 1e-31 | -72.280063 | 0.12% | 0.01% | motif file (matrix) |
| 3 | 0.924 | A C T G C A T G A G C T G T A C C G T A A G T C C A T G A G C T A C T G C G T A | 1e-30 | -70.923875 | 0.91% | 0.41% | motif file (matrix) |
